# Telomere-to-telomere reference genome for *Panax ginseng* highlights the evolution of saponin biosynthesis

**DOI:** 10.1093/hr/uhae107

**Published:** 2024-04-09

**Authors:** Yiting Song, Yating Zhang, Xu Wang, Xikai Yu, Yi Liao, Hao Zhang, Linfeng Li, Yingping Wang, Bao Liu, Wei Li

**Affiliations:** College of Plant Science and Technology, Huazhong Agricultural University, Wuhan 430070, China; Shenzhen Branch, Guangdong Laboratory for Lingnan Modern Agriculture, Shenzhen Key Laboratory of Agricultural Synthetic Biology, Genome Analysis Laboratory of the Ministry of Agriculture and Rural Affairs, Agricultural Genomics Institute at Shenzhen, Chinese Academy of Agricultural Sciences, Shenzhen 518124, China; Shenzhen Branch, Guangdong Laboratory for Lingnan Modern Agriculture, Shenzhen Key Laboratory of Agricultural Synthetic Biology, Genome Analysis Laboratory of the Ministry of Agriculture and Rural Affairs, Agricultural Genomics Institute at Shenzhen, Chinese Academy of Agricultural Sciences, Shenzhen 518124, China; National Key Laboratory of Tropical Crop Breeding, Shenzhen Branch, Guangdong Laboratory of Lingnan Modern Agriculture, Key Laboratory of Synthetic Biology, Ministry of Agriculture and Rural Affairs, Agricultural Genomics Institute at Shenzhen, Chinese Academy of Agricultural Sciences, Shenzhen 518124, China; Shenzhen Branch, Guangdong Laboratory for Lingnan Modern Agriculture, Shenzhen Key Laboratory of Agricultural Synthetic Biology, Genome Analysis Laboratory of the Ministry of Agriculture and Rural Affairs, Agricultural Genomics Institute at Shenzhen, Chinese Academy of Agricultural Sciences, Shenzhen 518124, China; College of Horticulture, South China Agricultural University, Guangzhou 510642, China; Institute of Special Animal and Plant Sciences, Chinese Academy of Agricultural Sciences, Changchun 130112, China; Ministry of Education Key Laboratory for Biodiversity Science and Ecological Engineering, Coastal Ecosystems Research Station of Yangtze River Estuary, Institute of Biodiversity Science and Institute of Eco-Chongming, School of Life Sciences, Fudan University, Songhu Road 2005, Shanghai 200433, China; State-Local Joint Engineering Research Center of Ginseng Breeding and Application, Jilin Agricultural University, Changchun 130118, China; Key Laboratory of Molecular Epigenetics of the Ministry of Education (MOE), Northeast Normal University, Changchun 130024, China; Shenzhen Branch, Guangdong Laboratory for Lingnan Modern Agriculture, Shenzhen Key Laboratory of Agricultural Synthetic Biology, Genome Analysis Laboratory of the Ministry of Agriculture and Rural Affairs, Agricultural Genomics Institute at Shenzhen, Chinese Academy of Agricultural Sciences, Shenzhen 518124, China; Kunpeng Institute of Modern Agriculture at Foshan, Shenzhen Branch, Guangdong Laboratory of Lingnan Modern Agriculture, Agricultural Genomics Institute at Shenzhen, Chinese Academy of Agricultural Sciences, Shenzhen 518124, China

## Abstract

Ginseng (*Panax ginseng*) is a representative of Chinese traditional medicine, also used worldwide, while the triterpene saponin ginsenoside is the most important effective compound within it. Ginseng is an allotetraploid, with complex genetic background, making the study of its metabolic evolution challenging. In this study, we assembled a telomere-to-telomere ginseng reference genome, constructed of 3.45 Gb with 24 chromosomes and 77 266 protein-coding genes. Additionally, the reference genome was divided into two subgenomes, designated as subgenome A and B. Subgenome A contains a larger number of genes, whereas subgenome B has a general expression advantage, suggesting that ginseng subgenomes experienced asymmetric gene loss with biased gene expression. The two subgenomes separated approximately 6.07 million years ago, and subgenome B shows the closest relation to *Panax vietnamensis var. fuscidiscus*. Comparative genomics revealed an expansion of gene families associated with ginsenoside biosynthesis in both ginseng subgenomes. Furthermore, both tandem duplications and proximal duplications play crucial roles in ginsenoside biosynthesis. We also screened functional genes identified in previous research and found that some of these genes located in colinear regions between subgenomes have divergence functions, revealing an unbalanced evolution in both subgenomes and the saponin biosynthesis pathway in ginseng. Our work provides important resources for future genetic studies and breeding programs of ginseng, as well as the biosynthesis of ginsenosides.

## Introduction

Ginseng (*Panax ginseng*) is one of the most important medicinal plants, grown in northeast Asia including China, Korea, Russia, and Japan, as well as a small amount in North America [1]. As recorded in the ancient Chinese book *Shen-nong’s Herbal Classics*, Ginseng’s perennial root has been used for several thousand years as traditional medicine, as well as a functional food and beverage, which has physical and immunological enhancement functions [2]. Ginseng was collected in the wild in fields for a very long history and its cultivation commenced about 500 years ago, since when breeding started and cultivars were generally formed [3, 4]. Due to the long growth cycle of ginseng, the polyploidy nature of its genome, and the ambiguity of phenotype, ginseng breeding is still in the preliminary stage and the germplasm is unstable during agricultural application.

The reference genome provides information for functional genes, evolution, and breeding, but a small number of medicinal plants have been sequenced and assembled to high-quality genomes [5, 6]. The innovations in sequencing technology make the assembly of more high-quality medicinal plant genomes possible [7, 8]. Two raw ginseng genome databases have been released using second generation sequence technology (Illumina platform), with an N50 value of 22 kb and 569 kb each, respectively [9, 10]. In our previous work, a better quality genome of ginseng (ginseng v1.0) is obtained by a nanopore platform with N50 = 19.75 Mb [11]. However, there are still several gaps that have not been filled. Based on ultra-long read sequencing technology with high-quality assembly tools, it is expected that these unknown regions will be read [12]. Further assembly of the telomere-to-telomere reference ginseng genome can serve as a more robust research foundation for the study of ginseng evolution, functional genes, and breeding.

Many medicinal plants are polyploid with increased research difficulties compared to normal crops and horticulture plants. In previous studies, fluorescence in situ hybridization (FISH) of ginseng revealed the PgDel2 elements signals showing an intensity bias toward 24 out of the 48 chromosomes, while unique gene probes further demonstrated two pairs of signals have different locations of chromosomes, collectively providing evidence for the allotetraploidy of ginseng [13]. Allotetraploids are formed by hybridisation and doubling of two or more different diploid species. Although doubling events are beneficial for enhancing species diversity and environmental adaptation, in the early stages of heteropolyploid formation, in order to co-ordinate and co-exist with the genomes from differently evolved diploid ancestor species in a single nucleus, the polyploid genomes undergo gene loss or divergence, or even chromosome reduction, obtaining a state more like that of the diploid [14, 15]. Previous research indicates the presence of subgenome dominance in heterologous polyploids, where one subgenome typically exhibits more genes, higher gene expression, and lower divergence [16–18]. Allele recombination in polyploidy cotton (*Gossypium hirsutum*) subgenomes contributes to ecological adaptation and fiber evolvement [19]. Rapid genomic change and homoeologous suppression leading to diploidization were also explored in wheat (*Triticum aestivum*) subgenome [20]. Clarifying the difference between subgenomes of ginseng will also help function and evolution studies.

Like other herbs, medicinal ginseng has complex metabolites that are considered effective compounds, among which triterpene saponin (ginsenoside) is the most important type. There are probably over a hundred ginsenosides in ginseng, but the synthetic pathways of the vast majority of ginsenosides remain unelucidated. The structure of ginseng saponin is a C30 backbone with oxidation and glycosylation in different sites. Ginseng mainly accumulates three types of saponin: protopanaxadiol (PPD), protopanaxatriol (PPT), and oleanane (OA) [21]. Ginsenosides are synthesized through a three-stage pathway [22, 23]. In the first stage, acetyl-CoA, a product of glycolysis, serves as the primary donor. It is used to synthesize isopentenyl diphosphate (IPP) and dimethylallyl diphosphate (DMAPP) via the intermediate metabolite mevalonate. In the second stage, isoprenyl diphosphate synthase and terpene cyclases convert IPP and DMAPP to 2,3-oxidosqualene. For the final stage, 2,3-oxidosqualene experiences complex modifications, including hydroxylation, cyclization, and glycosylation.

Catalyzed by oxidosqualene cyclases (OSCs), cytochrome P450 monooxygenases, and glycosyltransferases, the synthetic pathway leads to the formation of dammarane-type and oleanane-type ginsenosides. The biosynthetic pathway was partially elucidated while upstream enzymes were identified such as a 3-hydroxy-3-methylglutaryl-coenzyme A reductase (PgHMGR1 [24]), the key enzyme to generate common terpenoid precursor DMAPP and IPP, a farnesyl diphosphate synthase (PgFPS [25]) for C15 backbone formation, a squalene synthase (PgSS1 [26]) and a squalene epoxidase (PgSQE1 [27]) working for the biosynthesis of 2,3-oxidosqualene (the common precursor of triterpene). Then the pathway divided into different branches due to four oxidosqualene cyclases (OSCs): PNY1 [28] and PNY2 [29] as β-amyrin Synthase, PNA [30] and PgDDS [31] as dammarenediol synthase, leading to oleanane and PPT or PPD type ginsenoside respectively. Three CYP450s were reported for further oxidation: CYP716A52 [32] for 28-hydroxylation of β-amyrin, CYP716A53v2 [33] for 6-hydroxylation for PPT formation, and CYP716A47 [34] for 12-hydroxylation for PPD formation. Twelve glycosyltransferases were identified, e.g., PgUGT94Q2 for 3-glucosylation and UGTpg101 [35] for 6-glucosylation, other glucosyltransferases working to generate di- or tri-saccharide by transfer a glycosyl group to another; however, further glycosyl modifications are still to be clarified [21, 36]. Many biosynthesis genes in the same pathway are linearized in the plant genome forming metabolic gene clusters (MGC), especially for triterpene saponins [22, 23]. MGCs for saponin are also reported in medicinal plants such as *Salvia miltiorrhiza* [23] but have not been detected in Ginseng so far.

In this study, we assembled the telomere-to-telomere ginseng reference genome. Using this T2T reference genome, we studied the phylogeny and evolution of the ginseng genome, along with examining the asymmetric loss and biased expression of genes between subgenomes. Notably, genes involved in proximal duplication (PD) and tandem duplication (TD) are important for ginsenoside synthesis. We further explored the divergence of ginsenoside synthesis genes between subgenomes and identified gene clusters associated with ginsenoside synthesis. This reference genome enhanced our understanding of the evolution of saponin biosynthesis in allotetraploid ginseng and further facilitates genetics research and breeding of ginseng.

## Results

### *P. ginseng* reference genome assembly and annotation

**Figure 1:**
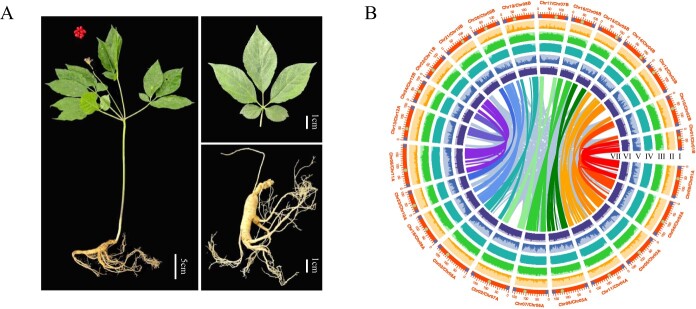
The morphological and genomic landscape of *Panax ginseng*. **A** Overview of the morphological characteristics of *P. ginseng*. **B** Genomic features of *P. ginseng*: circos plot from the outer to the inner circle represents chromosome-scale pseudochromosomes, blue circles indicate telomeres, green circles indicate centromeres (I), Copia density (II), Gypsy density (III), TE density (IV), gene density (V), GC content (VI), gene collinearity connected by curved lines (VII).

Our ginseng genome was assembled from data obtained through multiple sequencing platforms, including ~140G HiFi long reads, ~250G Oxford Nanopore Technologies (ONT) reads, ~140G Illumina read pairs, and ~ 700G Hi-C short reads. HiFi long reads and Hi-C short reads were used to assemble the initial ginseng chromosomes. Then TGS-GapCloser [37] was employed for initial gap filling and the remaining gaps were further filled by Graph-Based Gap Filling (GBGF) [38], resulting in 286 gaps filled (Fig. S1, see online supplementary material). Finally, Illumina reads were used to polish the genome. The ultimately assembled genome consisted of 24 contigs, with a contig N50 of 147.37 Mb (Fig. 1B; Fig. S2 and Table S1, see online supplementary material).

The assembled genome had a size of 3.45 Gb and a GC content of 34.71%, values that were larger than those of ginseng v1.0 [11] (Table 1). The quality assessment resulted in a QV value of 41.86, a K-mer completeness of 97.4025%, and Benchmarking Universal Single-Copy Ortholog (BUSCO) completeness of 99.3% (Table 1). These results are consistent with published telomere-to-telomere genomes, such as strawberry [39] and grape [40]. To further assess the fidelity of the T2T ginseng reference genome, we mapped RNA-seq paired-end reads to the genome, achieving a mapping rate of 94%–98%. In order to characterize whether the telomeres and centromeres of the ginseng genome are complete, we used quarTeT [41] and T2Tools (https://github.com/sc-zhang/T2Tools) for telomeres and centromeres predictions, respectively, and the results showed that there are a total of 48 telomeres and 24 centromeres (Fig. 1 and Table 1; Fig. S3 and Tables S1 and S2, see online supplementary material). In addition to the conventional telomeric sequence (AAACCCT)n, the ginseng genome has a special telomeric sequence (AAATTTT)n, located on chromosome 1 (Chr01). In brief, we successfully assembled a telomere-to-telomere ginseng reference genome.

**Table 1:** *Panax ginseng* genome assembly statistics

	*P. ginseng* vT2T	*P. ginseng* v1.0
Genome size (Gb)	3.45	3.36
GC content (%)	34.71	34.25
Number of contigs	24	425
N50 contig length (Mb)	147.37	19.75
Number of protein-coding genes	77 266	65 913
Repeat sequence content (%)	83.12	79.61
LTR content (%)	74.93	76.53
Gypsy content (%)	47.07	46.9
Copia content (%)	5.64	5.35
Number of telomeres	48	\
Number of centromeres	24	\
Quality value (QV)	41.86	\
K-mer completeness (%)	97.4	\
Complete genome BUSCO (%)	99.3	\
Complete gene prediction BUSCO (%)	98.4	95.14
LTR Assembly Index (LAI)	8.71	7.13

Transposal elements (TEs) annotation of the reference genome showed that the ginseng genome contains 83.17% of the genome in repeat sequences, similar to the found in maize genome [42]. Long terminal repeats (LTRs) are the most abundant in repeat sequences, accounting for 74.93% of the ginseng genome, of which LTR/*Gypsy* and LTR/*Copia* accounted for 47.07% and 5.64% of the genome, respectively (Table 1; Table S3, see online supplementary material). The distribution of repeat sequences across chromosomes is not uniform, and their content is lower towards the ends of the chromosomes (Fig. 1B). The LTR Assembly Index (LAI) for the ginseng genome was estimated to be 8.71, which is an improvement from the previous version (Table 1). The ginseng genome was annotated by combining *ab initio* prediction, homology-based searches and RNA-seq data. The final annotation yielded 77 266 protein-coding genes assessed for completeness of 98.4% according to BUSCO, which also is higher than that of the previous version of completeness (Table 1). The distribution of gene density on chromosomes is uneven, with a greater concentration observed at the chromosomal ends (Fig. 1B). After annotation, we found that out of the 286 filled gaps, 13 were located at telomeres, one at centromere, 22 at protein-coding genes, and 250 at repeat sequences (Fig. S1, see online supplementary material).

### Phylogenomic and evolution of *P. ginseng*

Given ginseng is an allotetraploid plant [13], we applied 13 K-mer based clustering and divided the ginseng chromosomes into two subgenomes (Fig. S4A, see online supplementary material) with good covariance (Fig. 1B). The size of subgenomes A and B are 1.94 Gb and 1.51 Gb, with 40 550 and 36 716 genes annotated, respectively. The subgenome-specific LTR-RTs insertion times of the two subgenomes were estimated, noting that subgenome A has earlier insertions compared to subgenome B (Fig. S4B, see online supplementary material). The difference in subgenome-specific LTR-RTs insertion time indicated that our subgenome sorting method was reasonable.

**Figure 2:**
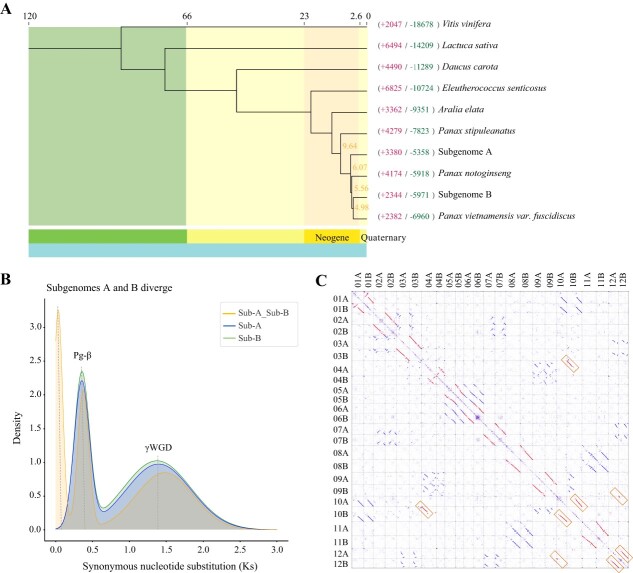
Comparative genomic analyses in *Panax ginseng* and other species. **A** Phylogenetic tree of ten species. Expansion and contraction of gene families are indicated in red and green, respectively. Numbers on the nodes represent the divergence time of the species (million years ago, MYA). **B** Synonymous substitution rate (Ks) distributions of subgenome A and subgenome B (Sub-A_Sub-B), subgenomes A (Sub-A) and subgenomes B (Sub-B). **C** Dot plot visualization of collinearity between 24 chromosomes. Yellow boxes indicate chromosomal rearrangements.

To study the evolutionary history of the ginseng and its subgenomes, we constructed two distinct phylogenomic trees (Fig. 2A; , see online supplementary materialFig. S5). The first tree included *Araliaceae* family plants, such as *Eleutherococcus senticosus*, *Aralia elata*, *Panax stipuleanatus*, *Panax vietnamensis var. fuscidiscus*, *Panax notoginseng*, *Panax quinquefolius*, *Panax japonicus*, *P. ginseng*, and *Vitis vinifera*, *Lactuca sativa*, and *Daucus carota*. The result showed that ginseng is most related to *P. japonicus*, separated by about 6.74 million years ago (Fig. S5, see online supplementary material). To further trace the origin of ginseng allotetraploid, we excluded tetraploids from the *Panax* genus (*P. quinquefolius* and *P. japonicus*) and constructed an evolutionary tree with two individual subgenomes of ginseng and eight species. Ginseng subgenome B is most closely related to *P. vietnamensis var. fuscidiscus*, diverging at about 4.98 million years. On the other hand, subgenome A separated from the common ancestor of *P. vietnamensis var. fuscidiscus, P. notoginseng* and subgenome B about 6.07 million years ago (Fig. 2A).

Subgenome A had 3380 expanded gene families and 5358 contracted gene families, whereas subgenome B had 2344 expanded and 5971 contracted. We performed KEGG enrichment analysis on both subgenomes A and B, which showed that the expanded gene families of subgenomes A and B were associated with sesquiterpene and triterpene biosynthesis. Moreover, the expanded gene family of subgenome A was also associated with cytochrome P450, suggesting the specific expansion of the gene families in subgenomes A and B to enrich the triterpene saponin products (Fig. S6, see online supplementary material).

To investigate the whole-genome duplication (WGD) events that occurred during the evolution of ginseng, we examined the distribution of Ks between homologous genes in two subgenomes of ginseng (Fig. 2B). Both subgenome A and subgenome B exhibit two distinct peaks around 0.35–0.38 and 1.37–1.48, indicating that both ginseng subgenomes shared the two genome-wide duplication events. Specifically, these events include γ-WGT shared by core-eudicot and lineage-specific duplications pg-β, respectively, consistent with previous reports [11]. The Ks distribution of the homologous genes between subgenomes A and B demonstrated specific peaks at about 0.03, but the peak was absent in subgenome A or subgenome B, implying the divergence between the two subgenomes. The estimated divergence time is approximately 6.07 million years (Fig. 2A and B).

A comprehensive collinearity analysis of homologous chromosomes was performed, unveiling three distinct collinearity patterns (Fig. 2C). Firstly, a pronounced collinearity was observed between corresponding chromosomes of the two subgenomes, such as Chr11A and Chr11B, Chr08A and Chr08B, indicating a relatively complete chromosome structure that has diverged from a common progenitor during their evolutionary history. Furthermore, significant collinearity within subgenomes was identified, such as Chr11B and Chr08B, Chr11A, and Chr08A, implying that both subgenomes have undergone a WGD event. Additionally, by examining the dot plots, it was observed that Chr10B was covariant with both Chr04A and Chr10A, while Chr12B was covariant with both Chr10A and Chr12A, suggesting that chromosome rearrangements have occurred within the subgenome, indicating divergent evolutionary trajectories of the ginseng subgenomes.

Despite the clear collinearity between homologous chromosomes across subgenomes, their chromosome sizes varied considerably. Therefore, we performed a comparative analysis of homologous chromosomes between subgenomes and identified a large number of structural variations including 227 inversions, 10 867 translocations and 5354 duplications (Fig. S7 and Table S4, see online supplementary material). Notably, there are many particularly large regions of inversions accompanied by deletions, such as Chr01, Chr02, and Chr09 (Fig. S7, see online supplementary material). These structural variations may contribute to the observed differences in chromosome lengths between subgenome A and B. The identification of these structural variations provides valuable insights into the dynamic changes that have occurred during the evolutionary divergence of the ginseng subgenomes (Fig. S7, see online supplementary material). These variations provide preliminary information regarding their potential impact on biological functionality.

### Subgenomes A and B experienced asymmetric gene loss with biased gene expression

The distribution of functional genes between subgenomes A and B in the ginseng reference genome is not yet fully understood. It remains uncertain whether they undergo asymmetric gene deletions, exhibit gene expression bias, undergo specific natural selection, or share similarities with observations in other allopolyploid plants [43, 44]. To address these questions, we conducted a comparative analysis of the gene composition, expression patterns, and genetic diversity of subgenomes A and B.

**Figure 3:**
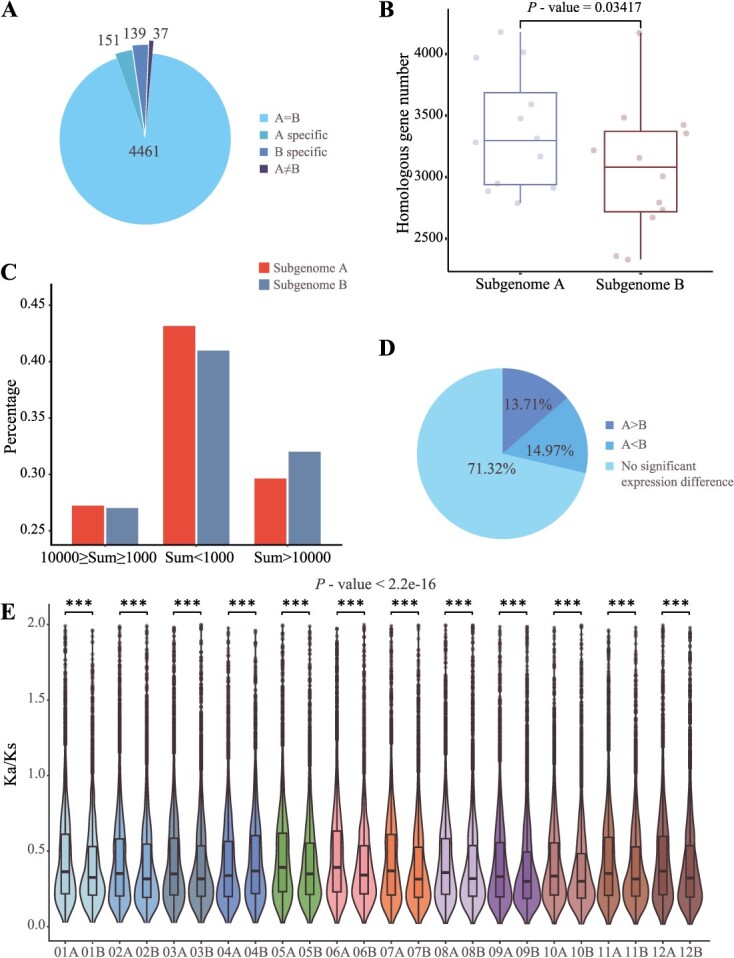
Subgenome dominance of *Panax ginseng* genomes. **A** Comparison of gene counts with each Pfam between subgenomes. ‘A specific’: only in subgenome A, ‘B specific’: only in subgenome B; ‘A = B’: no significant, ‘A ≠ B’: significantly different between A and B. **B** The difference in the number of chromosomal homologous gene between subgenomes. *Panax stipuleanatus* was employed as an outgroup. Wilcoxon test with a two-sided alternative hypothesis was applied. The box-and-whisker plot displays the median, upper/lower quartile, and interquartile range (IQR). The box spans IQR, with vertical lines indicating the 0.25 and 0.75 quartiles and the median is represented by a line inside the box. **C** Unequal expression of homologous gene from subgenomes. Three expression levels were divided from 10 RNA-seq datasets. **D** Percentage of syntenic gene pair in three expression catalogs. ‘A > B’: higher in subgenome A, ‘A < B’: higher in subgenome B (*P*-value > 0.05, | logFC | < 1). **E** Ka/Ks ratios of gene pairs originating from each chromosome of subgenomes A and B. The box-and-whisker plot displays the median, upper/lower quartile, and interquartile range (IQR). The box spans IQR, with vertical lines indicating the 0.25 and 0.75 quartiles and the median is represented by a line inside the box. The significance test was conducted using the Kruskal–Wallis test. Subgenome chromosomes exhibit significant differences (two-tailed Student's *t* test, *P* < 0.001).

As a crucial indicator of gene function, the number of genes with certain protein domains decreases along with gene loss in specific subgenomes. Pfam domains and their corresponding gene counts were compared between subgenomes A and B. Out of the 77 266 genes located on 24 chromosomes, 69 242 genes (89.62%) contained 4788 Pfam domains. This indicates a significant difference in the number of genes associated with this Pfam domain between the two subgenomes compared to the total number of genes in their respective subgenomes, with a *P*-value <0.05 in Fisher’s exact test. Fig. 3A illustrates that 3.15% (151/4788) and 2.90% (139/4788) of the Pfam domains were specifically present in subgenomes A and B, respectively, highlighting the differences in gene functions. Additionally, a significant difference was observed in the counts of retained homologous genes of *P. stipuleanatus* in the two subgenomes (*P*-value = 0.03417), as depicted in Fig. 3B. This provides further evidence that gene loss occurred after subgenome divergence.

We investigated the expression profiles of ginseng genes homologous to *P. stipuleanatus*. Based on the total read counts in the RNA-sequencing data, genes were grouped into three categories with distinct expressions: high, low, and middle expression. To compare the distribution of each group between intersubgenome homoeologous chromosomes, we analysed their percentages by taking the total number of homologous genes in each subgenome as the background. Interestingly, we observed that the percentages of homologous genes from subgenome A and B did not exhibit differences in groups with middle expression. However, in the low-expression group, the percentage of homologous genes in subgenome B was lower than that in subgenome A. On the contrary, in the high-expression group, the percentage of homologous genes in subgenome B was higher than that in subgenome A (Fig. 3C). Thus, the gene expression of subgenomes A and B may differ significantly.

We performed paired *t*-tests to assess the expression level of homologous genes from each subgenome. Differentially expressed gene pairs were examined by analysing 10 transcriptome data. Among the total of 33 426 homologous gene pairs analysed, the majority (71.32%, 23 839/33426) displayed comparable expression patterns in both subgenomes. However, 13.71% of the gene pairs (4583/33426) had significantly higher expression in subgenome A and 14.97% of the gene pairs (5004/33426) had significantly higher expression in subgenome B (Fig. 3D). This suggests that there is biased expression of genes in subgenomes A and B.

We conducted function enrichment analysis for the genes with higher expression in each subgenome. The results revealed that most of them are enriched in different pathways; for example, genes significantly highly expressed in subgenome A are enriched in the protein catabolic process (GO:0030163), protein phosphatases and associated proteins. Conversely, genes significantly highly expressed in subgenome B are enriched in cellular response to chemical stimulus (GO:0070887), small molecule metabolic process (GO:0044281) and metabolism (Fig. S8, see online supplementary material). These results suggest that the differential expression observed in homologous gene pairs may not be random.

Hybridization plays a crucial role in driving evolutionary processes while investigating the distinct selection pressures between two subgenomes following hybridization is necessary. To address this, we conducted a comparative analysis of the Ka/Ks values (nonsynonymous/synonymous substitution rate) of 12 pairs of chromosomes comparatively between the subgenomes A and B. It found that the Ka/Ks values for all chromosomes were lower than 1, indicating that both subgenomes have undergone purifying selection. However, closer inspection reveals that subgenome B generally experienced more stringent purifying selection than subgenome A in the remaining chromosomes, with the exception of Chr 04 (Fig. 3E).

### Different types of gene duplication promote the flourishing of biosynthetic genes for ginseng triterpenoid saponins

We identified a total of 37 186 duplicated genes in subgenome A and 33 235 in subgenome B, classified into five categories: dispersed duplication (DSD) genes (37.82% in sub-A and 33.34% in B), WGD genes (29.95% in subgenome A and 33.90% in B), transposed duplication (TRD) genes (24.25% in subgenome A and 24.13% in B), tandem duplication (TD) genes (3.93% in subgenome A and 4.68% in B), and proximal duplication (PD) genes (4.05% in subgenome A and 3.96% in B) (Fig. S9, see online supplementary material).

**Figure 4:**
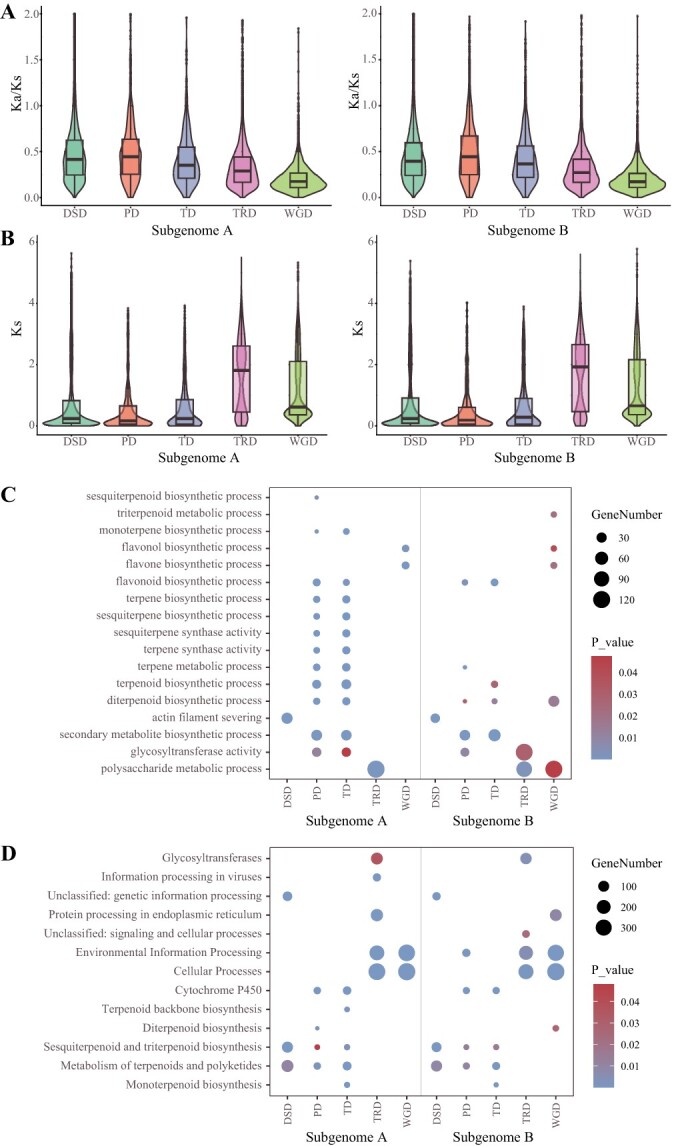
Gene duplication and evolution. (**A**) Ka/Ks ratios and (**B**) Ks values of gene pairs generated by different types of gene duplication. DSD, dispersed duplication; PD, proximal duplication; TD, tandem duplication; TRD, transposed duplication; WGD, whole-genome duplication. The box-and-whisker plot displays the median, upper/lower quartile, and interquartile range (IQR). The box spans IQR, with vertical lines indicating the 0.25 and 0.75 quartiles and the median is represented by a line inside the box. (**C**) GO and (**D**) KEGG enrichment analyses of genes originated from different gene duplication types. The enriched terms with *P*-value <0.05 are presented. The color of the bubbles indicates the statistical significance of the enriched terms; the size of the bubbles indicates the number of genes.

Ks and Ka/Ks values were compared across the two subgenomes among the groups of duplicated genes and were found to be largely consistent (Fig. 4A and B). Furthermore, when comparing different duplication modes, it is interesting to observe that DSD, TD, and PD genes had smaller Ks values compared to other types (Fig. 4B), indicating that DSD, TD, and PD genes are relatively recent duplications. Moreover, PD genes exhibited higher Ka/Ks ratios (Fig. 4A), suggesting that they have undergone more relaxed purifying selection. Similar conclusions are obtained in previous studies in *Angelica sinensis* [45] and *Cinnamomum camphora* [46].

To investigate the functional enrichment of duplicated genes, we conducted GO and KEGG analyses on different types of gene duplications (Fig. 4C and D), and the enrichment results observed some differences between subgenomes A and B. Firstly, the GO enrichment results for subgenome A showed that PD and TD are enriched in the biosynthesis process of various metabolites, such as terpenes, flavonoids, and glycosyltransferase activity (GO:0016757). TRD is mainly enriched in the polysaccharide metabolic process (GO:0005976), while WGD is enriched in the flavone biosynthetic process (GO:0051553). Then, from the GO enrichment results for subgenome B, PD and TD are enriched in the secondary metabolite biosynthesis process (GO:0044550), flavonoid biosynthesis process (GO:0016114). Furthermore, TD is enriched in terpenoid biosynthesis process (GO:0016114), and PD is enriched in glycosyltransferase activity (GO:0016757). In addition, TRD is mainly enriched in glycosyltransferase activity (GO:0016757) and polysaccharide metabolic process (GO:0005976). From the KEGG enrichment results for subgenomes A and B, the newly formed DSD, PD, and TD were enriched in sesquiterpenoid and triterpenoid biosynthesis, and PD and TD were enriched in cytochrome P450. Overall, these findings suggest that gene duplication has enriched the biosynthetic genes for important metabolites in ginseng. The recently formed TD and PD play critical roles in the emergence of plant secondary metabolites, particularly in triterpenoid saponin biosynthesis.

To further explore the role of TD and PD for gene expansion in triterpene saponin synthesis, we analysed OSC, CYP450, and UGT genes located relatively downstream in the pathway. The analysis revealed that 7.14% (2/28) and 10.71% (3/28) of OSC genes were classified as TD and PD genes, respectively. For CYP450 genes, 17.09% (94/550) and 21.45% (118/550) of them were expanded during TD and PD. Moreover, 16.12% (44/273) and 19.05% (52/273) of UGT genes were categorized as TD and PD genes, respectively. Among functional elucidated catalytic genes identified in ginsenoside biosynthesis, the UGT genes, one of the major factors causing the diversification of ginsenoside, are detected as TD or PD genes: UGT71A29 (pg_10003783) and pg_10003803, pg_10003804; UGTPg45 (pg_1002165) and pg_1002163; UGTPg100 (pg_11010095) and pg_11010100, pg_11010101; UGT71A27 (pg_4003612) and pg_4003638 are generated by PD; while UGTPg101 (pg_10003758) and pg_10003757; UGT94Q2 (pg_5001060) and pg_5001061 are generated by TD. This result further supports the hypothesis that the furnishing of triterpene saponin biosynthesis in ginseng was driven by gene duplication during evolution.

### The biosynthetic pathway of ginsenosides

**Figure 5:**
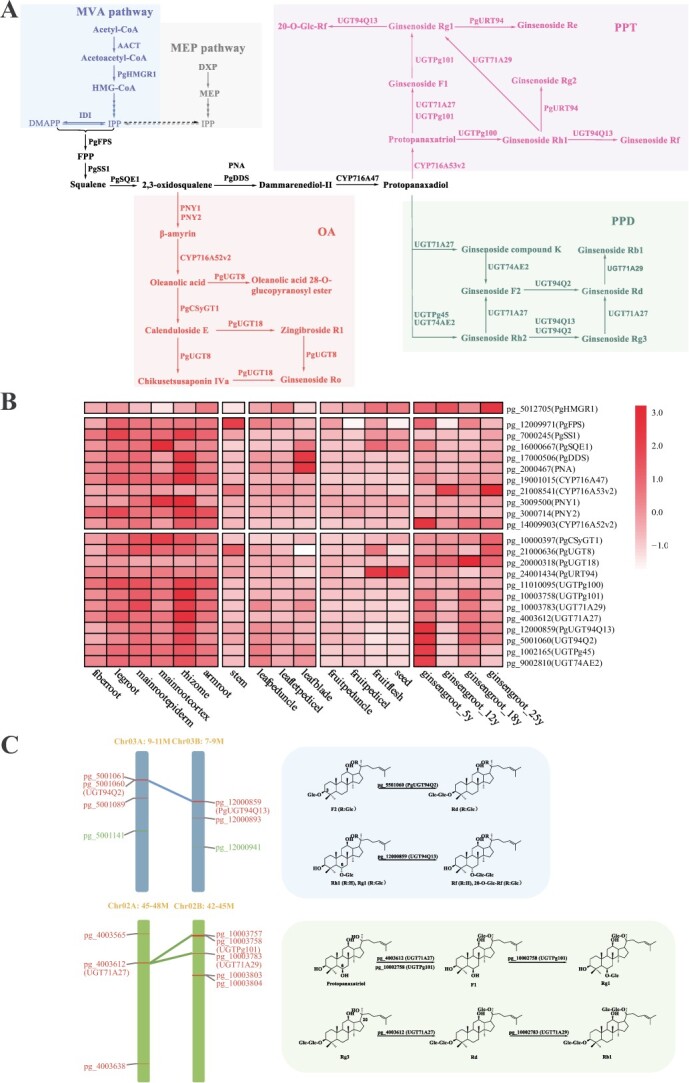
Metabolite pathway and heat maps of triterpenoid saponins biosynthesis pathways in *Panax ginseng*. **A** Metabolite pathway of triterpenoid saponins biosynthesis pathways in *P. ginseng*. **B** Heat map of the expression of genes involved in the triterpene saponin biosynthetic pathway in *P. ginseng*. ginsengroot_5/12/18/25y represents ginseng roots representing the years 5/12/18/25, respectively. **C** Chromosomal location map of homologous genes and types of catalytic reactions. UGT genes are shown in red font and CYP450 genes in green font.

There are more than 20 steps of catalytic steps that participate in the biosynthesis of ginsenosides, with key enzymes including scaffold formation squalene synthase (SS) and 2,3-oxidosqualene cyclase (OSC), as well as tailoring enzymes like cytochrome P450s (CYP450), and glycosyltransferases (UGT) [21]. By conducting gene function annotation and blastp analysis, we effectively identified 23 pivotal enzyme genes within this ginseng reference genome that are directly involved in the ginsenosides biosynthetic pathway and documented gene functions [48–50] (Fig. 5A). We have meticulously delineated their individual catalytic steps and thoroughly examined their expression patterns in 14 ginseng tissues and four vintages of ginseng roots, finding that P450 and UGT genes were mostly highly expressed in roots, especially rhizome (Fig. 5B).

The biosynthetic enzymes of saponins in ginseng also show divergence between subgenomes. We found several homologous genes in the colinear region of subgenomes located in different steps of the pathway (Fig. 5C). Pg_5501060 (UGT94Q2) on Chr3A and pg_12000859 (UGT94Q13) on Chr3B are homologous genes of subgenomes. However, Pg_5501060 (UGT94Q2) is responsible for the glycosylation of F2 at 3-Glc to 3-Glc-Glc, producing Rd in the PPD section [47], while pg_12000859 (UGT94Q13) glycosylates Rh1 and Rg1 at 6-Glc to 6-Glc-Glc, producing Rf and 20-O-Glc-Rf in the PPT section [48] (Fig. 5C). Additionally, Pg_4003612 (UGT71A27) on Chr02A has two corresponding genes on Chr02B: pg_10002758 (UGTPg101) and pg_10002783 (UGT71A29) which also have diverse functions. All three enzymes are capable of adding glucose group to 20-C, while pg_10002758 (UGTPg101) has an additional function of adding glucose to 6-C, and pg_10002783 (UGT71A29) can transform another glucose to 20-O-Glc [35, 47, 49].

We discovered 28 OSC genes, 273 UGT genes, and 550 CYP450 genes in ginseng (Fig. S9, see online supplementary material). Furthermore, through chromosomal mapping of OSC, UGT, and CYP450 genes, we have identified 13 triterpenoid saponin synthesis gene clusters in ginseng, which are located in the region near the telomeres. Remarkably, 12 of these clusters are symmetrically distributed on two subgenomes, although the copy number of enzyme genes varied between subgenomes (Figs S10 and S11, see online supplementary material).

## Discussion

Due to the short domestication history of medicinal plants, their genetic backgrounds are often scrambled, making genome mining challenging. Here, we assembled a 3.45 Gb telomere-to-telomere ginseng reference genome with BUSCO completeness of 99.3%, including 48 telomeres and 24 centromeres (Table 1). This reference genome facilitates the elucidation of the evolution of the allotetraploid *Panax* genus and the gene variations in the saponin biosynthetic pathway during genome evolution, which will guide further domestication of ginseng for enhanced medical effectiveness.

Based on phylogenomic analysis, we outline a scenario for the evolution of ginseng. The ancestral eudicot genome underwent two WGD events, i.e., γ-WGT (core-eudicot-shared) and Pg-β (lineage-specific duplication), leading to the formation of the ancestral *Panax* genome with 12 chromosomes. Two subgenomes of ginseng originated from this ancestral *Panax* genome and diverged around 6.07 million years ago. Subgenome B is more closely related to *P. vietnamensis var. fuscidiscus*, whereas subgenome A is the out group of clade *P. vietnamensis var. fuscidiscus*, *P. notoginseng* and subgenome B (Fig. 2). Those results show that ginseng should have originated from the hybridization between these two distinct species, which is similar to the formation of polypoid wheat (*T. aestivum*) [20].

Polyploids usually experience subgenome reconstruction with genes from different subgenomes expressed biasedly [43, 44]. Subgenome A has a relatively larger size compared to subgenome B, which has a lesser gene number. Pfam domains specifically present in subgenomes A and B (151 and 139, respectively) suggest potential differences in the distribution of domains in ginseng subgenomes. Although the two subgenomes show good collinearity (Fig. 1), their detailed structure has variations such as inversion and deletions. Subgenome A has more genes in the low-expression group, whereas subgenome B has more genes in the high-expression group. The number of homolog genes indicating higher expression in subgenome A (4,583) is lower than the genes with higher expression in subgenome B (5,004). The Ka/Ks values for both subgenomes are less than 1, meaning that both subgenomes have experienced purifying selection (Fig. 3).

Gene duplication is the main force for new gene formation and the furnishing of plant specialized natural products. Ka/Ks analyses revealed that DSD, TD, and PD genes were formed relatively recently, and PD genes, in particular, have undergone more relaxed purifying selection (Fig. 4). Notably, PD and TD increased the copy number of triterpene saponin synthesis pathway genes, with approximately 35.16% (96/273) of UGT genes belonging to PD or TD, including six genes that have been functionally validated. OSCs and CYP450s also underwent duplications, 7.14% PD and 10.71% TD for OSCs, and 17.09% PD and 21.45% TD for CYP450s respectively, suggesting the crucial roles of PD and TD in the evolution of saponins biosynthesis.

From this new reference genome version, we also found subgenome-specific genes with diversified functions. Colinear genes from corresponding subgenomes may participate in different roles in ginsenoside biosynthesis pathways. All this evidence indicates that both subgenomes have experienced functional diversification, which may benefit the survival and reproduction of gensing [34]. We also found several possible metabolic gene clusters in this genome that may be involved in saponin biosynthesis, and further experimental studies are necessary.

In summary, a telomere-to-telomere reference genome was assembled for allotetraploid ginseng, and then the relationship between polyploidization, subgenome dominance, and the biosynthesis of natural product saponin ginsenosides is explored. We believe this golden genome will be useful for ginseng germline selection and push the breeding of medicinal plants to further generations.

## Materials and methods

### Genome sequencing and assembly

Ginseng from Dandong, Jilin Province, China was collected as the sequencing material. Fresh roots were used for DNA extraction and sequencing.

HiFi long reads were assembled via hifiasm [50]. The assembled contigs were aligned to the reference genome (ginsengv1.0) using RagTag [51] for initial positioning and ordering. Subsequently, Hi-C scaffolding was performed using Juicer [52] and 3D-DNA [53] v180114 pipeline. Manual adjustments and error correction were carried out by utilizing Juicebox Assembly Tools (https://github.com/aidenlab/Juicebox) to generate the chromosome-level ginseng genome. Oxford Nanopore Technologies (ONT) reads (SRR16036174–213) were corrected using NextDenovo [54]. The corrected ONT reads were then used for the first round of gap filling on the genome, using TGS-gapcloser [37]. The remaining gaps were further filled by the Graph-Based Gap Filling (GBGF) [38]. Finally, the genome was polished using Merfin [55]. Benchmarking Universal Single-Copy Orthologs (BUSCO [56] v5.2.2) and the LTR Assembly Index (LAI [57]) were used to evaluate the assembly quality of the chromosome-level genome.

### Genome annotation

Transposable elements were identified using the EDTA [58] v1.9.4 pipeline. Coding gene structures were predicted through the Geta pipeline (https://github.com/chenlianfu/geta), incorporating *ab initio* predictions, homolog proteins, and transcriptome data (SRA accession: SRR16036220–29, SRR13131364–405, SRR2952867–84). The functional annotation of ginseng proteins was accomplished using eggnog-mapper [59] v5.0.2. Telomeres and centromeres were predicted employing quarTeT [41] and T2Tools (https://github.com/sc-zhang/T2Tools). The ginseng genome was separated into subgenomes A and B using the SubPhaser [60].

### Phylogenetic analyses

OrthoFinder [61] v2.5.5 was utilized to identify paralogs and orthologs and to infer the species tree among 11 plant species: *E. senticosus* [62], *A. elata* [63], *P. stipuleanatus* [11], *P. vietnamensis var. fuscidiscus* [64], *P. notoginseng* [65], *P. quinquefolius* [11], *P. japonicus* [11], *P. ginseng*, *V. vinifera* [66], *L. sativa* [67], and *D. carota* [68]. The species tree served as an input for estimating divergence time using the MCMCTree program within the PAML [69] package. Multiple fossil times from TimeTree (http://www.timetree.org/) were incorporated for time calibrations. Gene family expansion and contraction were inferred using CAFE5 [70] based on the chronogram of the aforementioned 11 plant species.

### Syntenic analyses

BLASTP was used to identify homologous genes both within and between species, and MCscanX [71] defined synteny blocks based on these identified homologs. Ks density for both paralogous and orthologous gene pairs was calculated using WGDI [72], and WGDI was additionally employed to generate the synteny dotplots. Structural variations between subgenomes were detected using SyRI [73].

### Gene duplication identification

Utilizing DupGen Finder [74] v1.12, the duplicated genes in ginseng were categorized into five groups: WGD, TD, PD, TRD, and DSD. Subsequently, genes within these duplicate categories underwent further GO and KEGG analyses using the R package clusterProfiler [71] v4.0. KaKs_Calculator [75] was employed for calculating the values of Ka (nonsynonymous substitutions per nonsynonymous site) and Ks (synonymous substitutions per synonymous site).

### Gene expression and functional analysis

The raw RNA-seq data (SRR16036220–29, SRR13131364–405, SRR2952867–84) underwent filtering using FASTp [76] v0.20.1. Subsequently, the filtered data were aligned to the ginseng genome using HISAT2 [77] v2.1.0, and transcript assembly and gene expression analysis were conducted using StringTie [78] v2.1.3b.

## Acknowledgements

We thank Dr Jianbin Yan for comments and advice; Dr Guiqi Bi and Huan Wang for guiding bioinformatics analysis. This work is supported by the National Key R&D Program of China (grant nos 2020YFA0907900 and 2022YFD1700200), Agricultural Genomics Institute at Shenzhen (AGIS-ZLXM202204), Science and Technology Development Project of Jilin Province (20210509022RQ).

## Author contributions

W.L. conceived and designed the study with B.L., Y.W., L.L., H.Z., and Y.L. Y.Z. prepared the materials. Y.S. performed the bioinformatic analyses. X.Y. and X.W. assisted in bioinformatics analyses. Y.S. and W.L. wrote the manuscript. All authors read and approved the final manuscript.

## Data availability

RNA-seq data and ONT data were obtained from the NCBI BioProjects database under accession numbers PRJNA752920 and PRJNA302556. HiFi data, Hi-C data, Illumina data, genome assembly data have been deposited in the China National Center for Bioinformation (https://www.cncb.ac.cn/) under BioProject number PRJCA022032. Genome annotation files are stored at https://doi.org/10.6084/m9.figshare.25477741.v1.

## Conflict of interest statement

The authors declare that they have no conflicts of interest.

## Supplementary data

Supplementary data is available at *Horticulture Research* online.

## Supplementary Material

Web_Material_uhae107
