# Supplementary material for: Telomere-to-telomere reference genome for Panax ginseng highlights the evolution of saponin biosynthesis
Source: Hortic Res. 2024 Apr 9;11(6):uhae107. doi: 10.1093/hr/uhae107 (PMC11179851; doi:10.1093/hr/uhae107)
Supplement: Web_Material_uhae107 [file web_material_uhae107.zip › sfig.pdf]

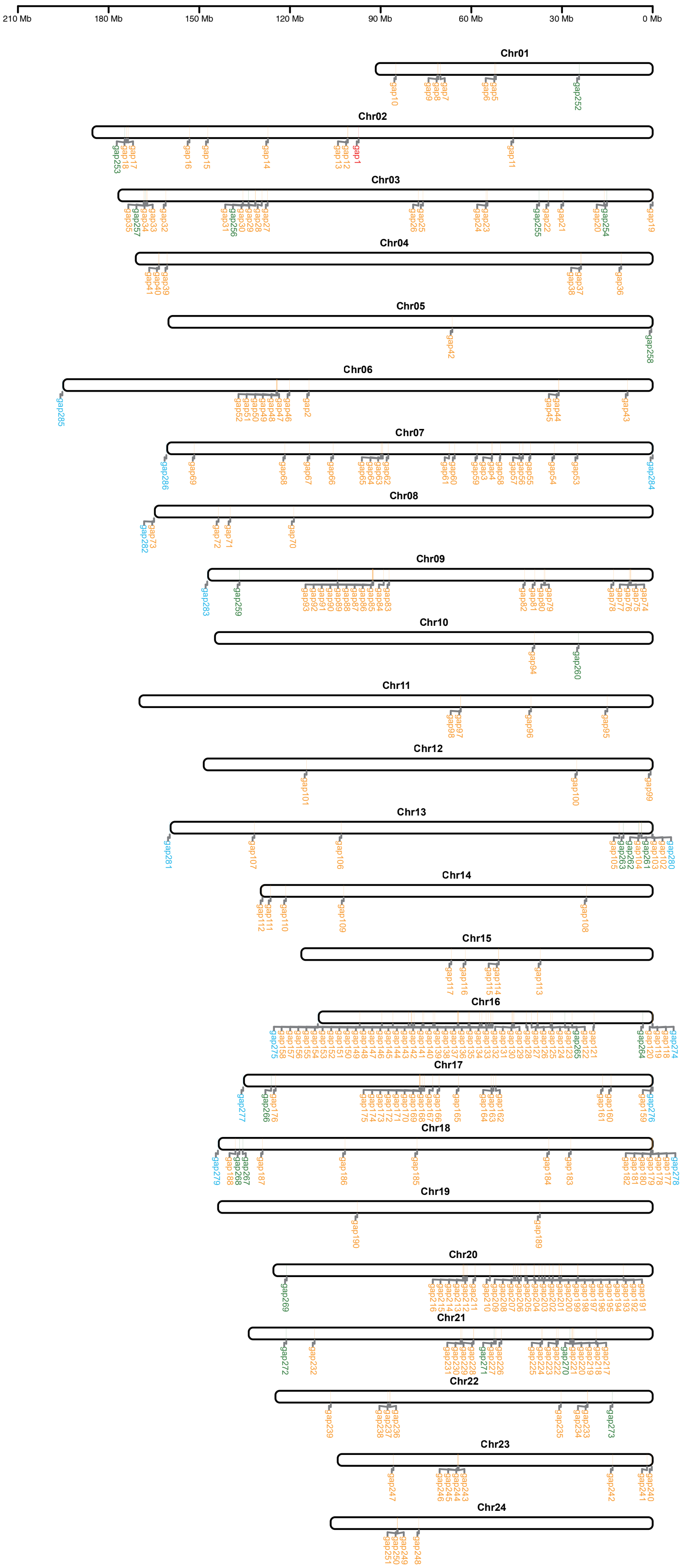

Supplementary Figure 1. Chromosomal localization maps of Panax ginseng genome-filled gaps. The font colors represent different regions on the genome, yellow for repeat sequences, green for protein coding genes, blue for telomeres and red for centromeres.

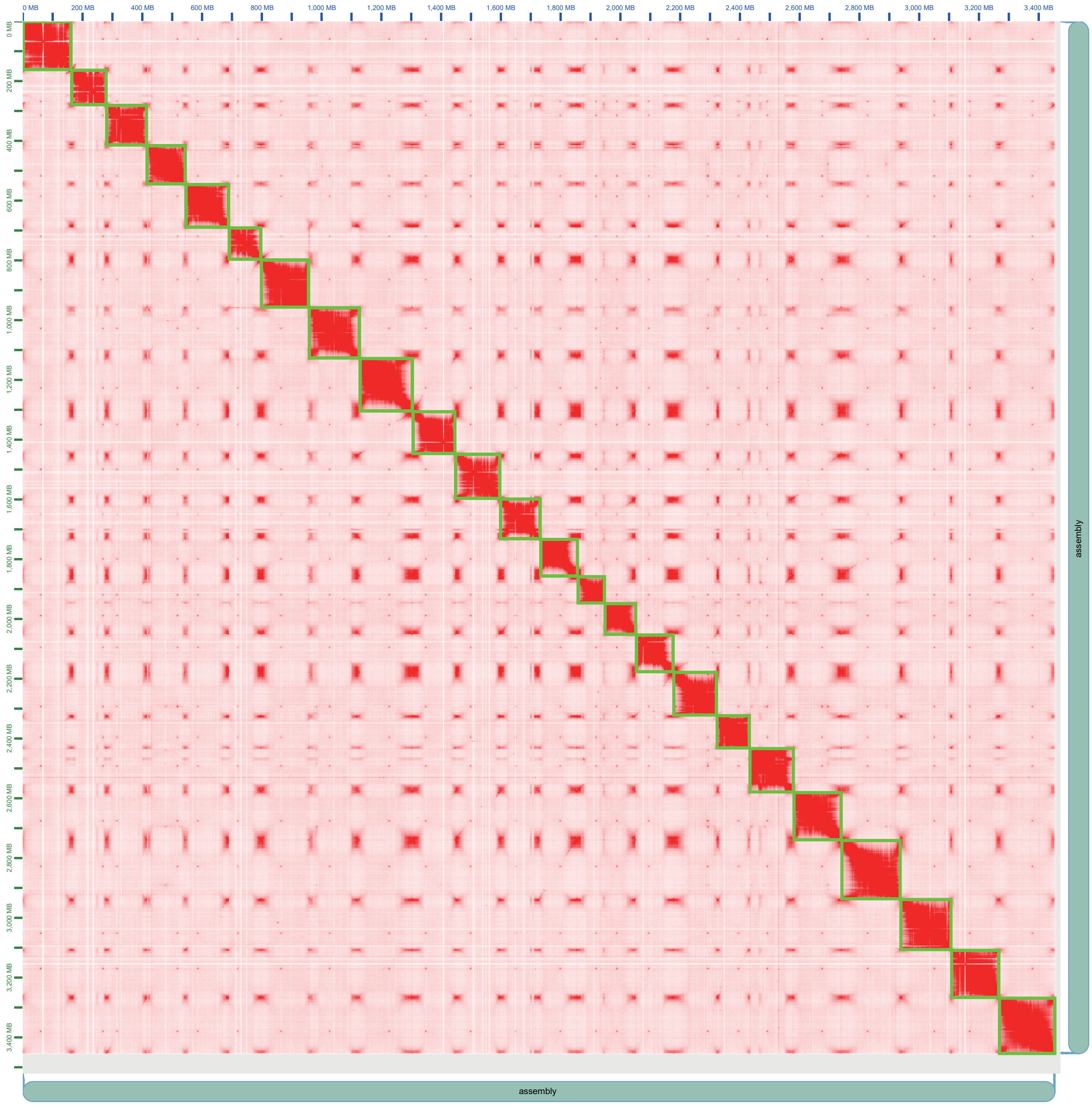

Supplementary Figure 2. Heatmap of chromatin contact matrices generated by aligning a Hi-C dataset to the Panax ginseng genome.

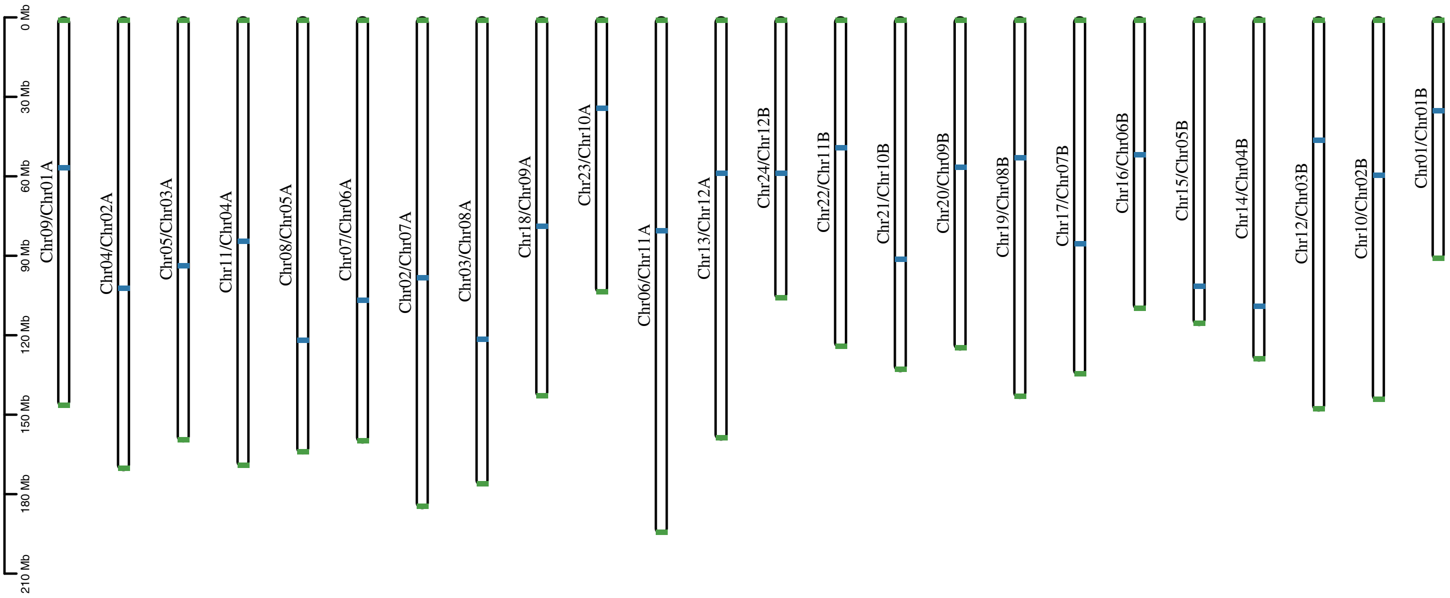

Supplementary Figure 3. Telomere and centromere detection map. green color indicates telomere; blue color indicates centromere.

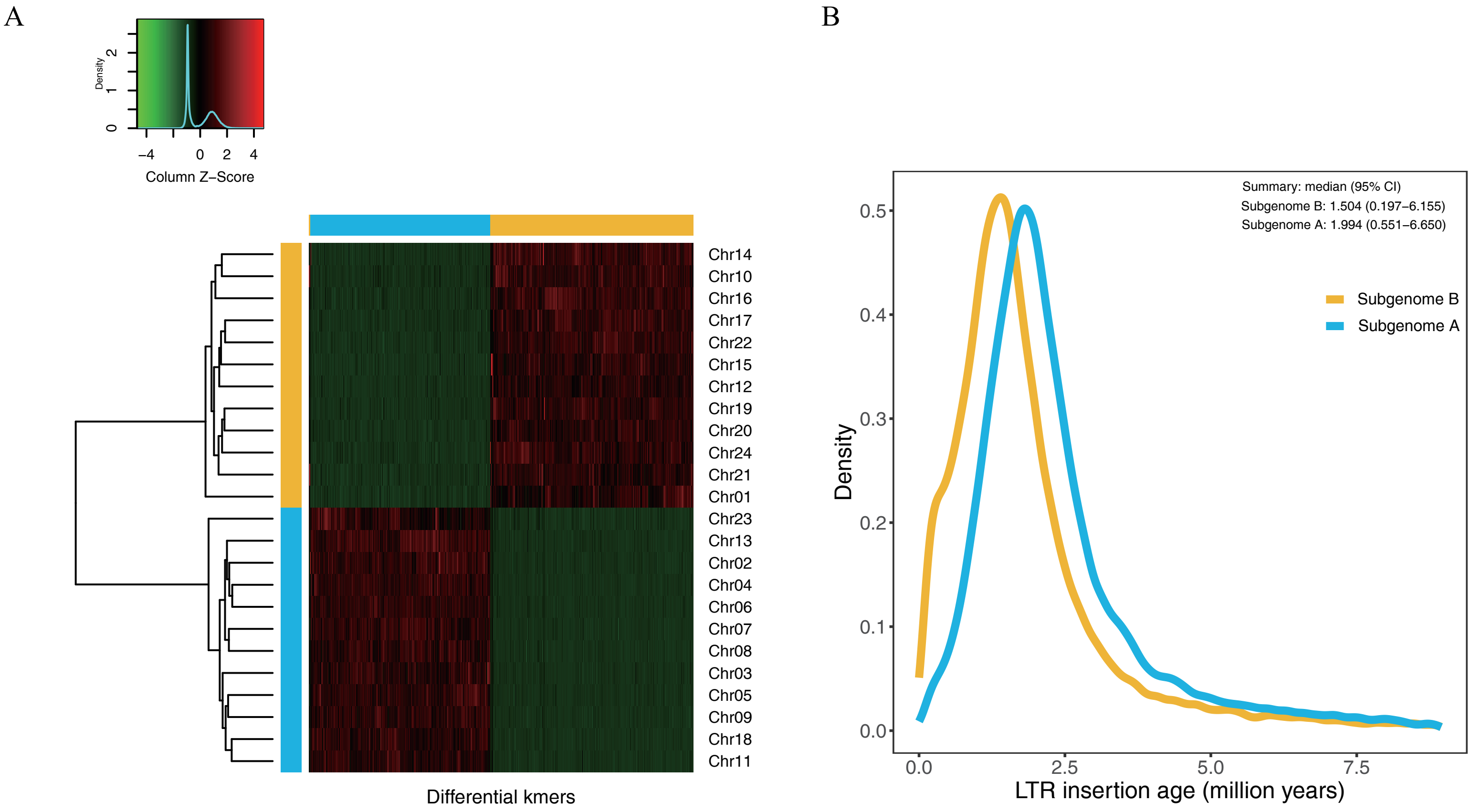

Supplementary Figure 4. (A) Heatmap and clustering of differential k-mers. The x-axis, differential k-mers; y-axis, chromosomes. The vertical color bar, each chromosome is assigned to which subgenome; the horizontal color bar, each k-mer is specific to which subgenome (blank for non-specific k-mers). (B) Insertion time of subgenome-specific LTR-RTs.

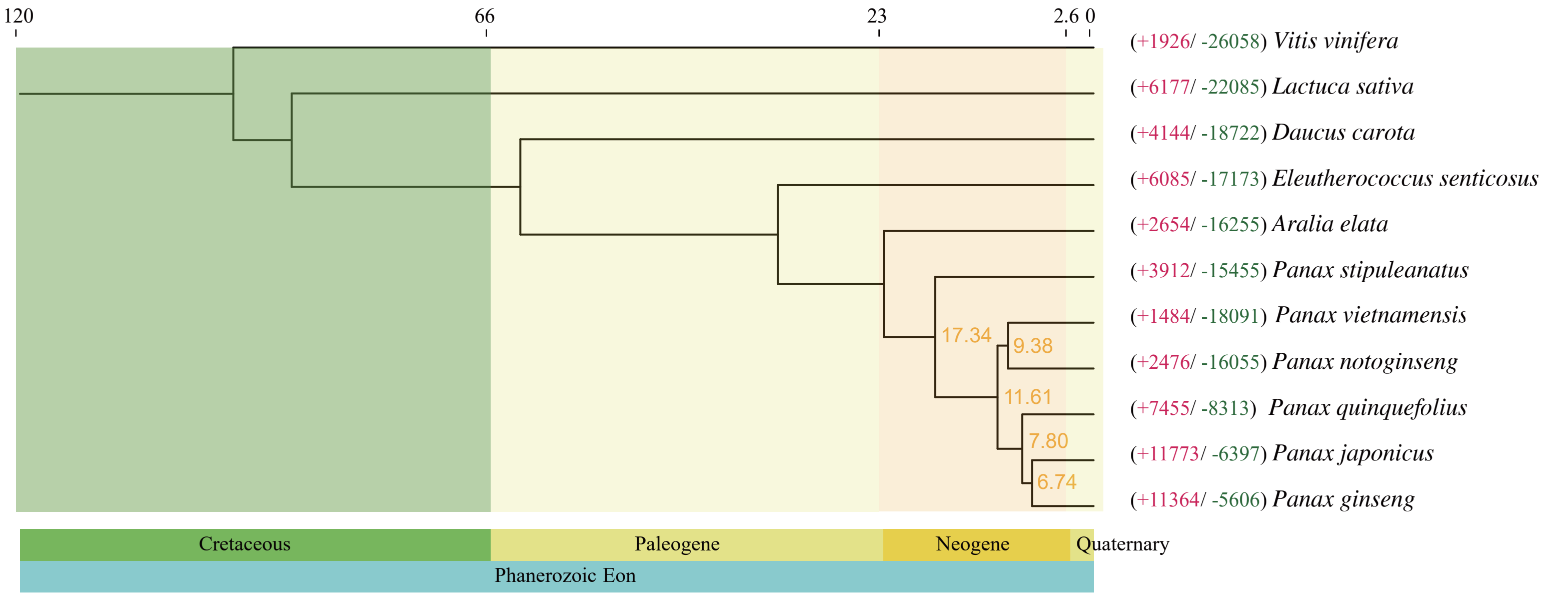

Supplementary Figure 5. Phylogenetic tree of eleven species. Expansion and contraction of gene families are indicated in red and green, respectively. Numbers on the nodes represent the divergence time of the species (million years ago, MYA). ***Panax vietnamensis*** is ***Panax vietnamensis var. fuscidiscus***.

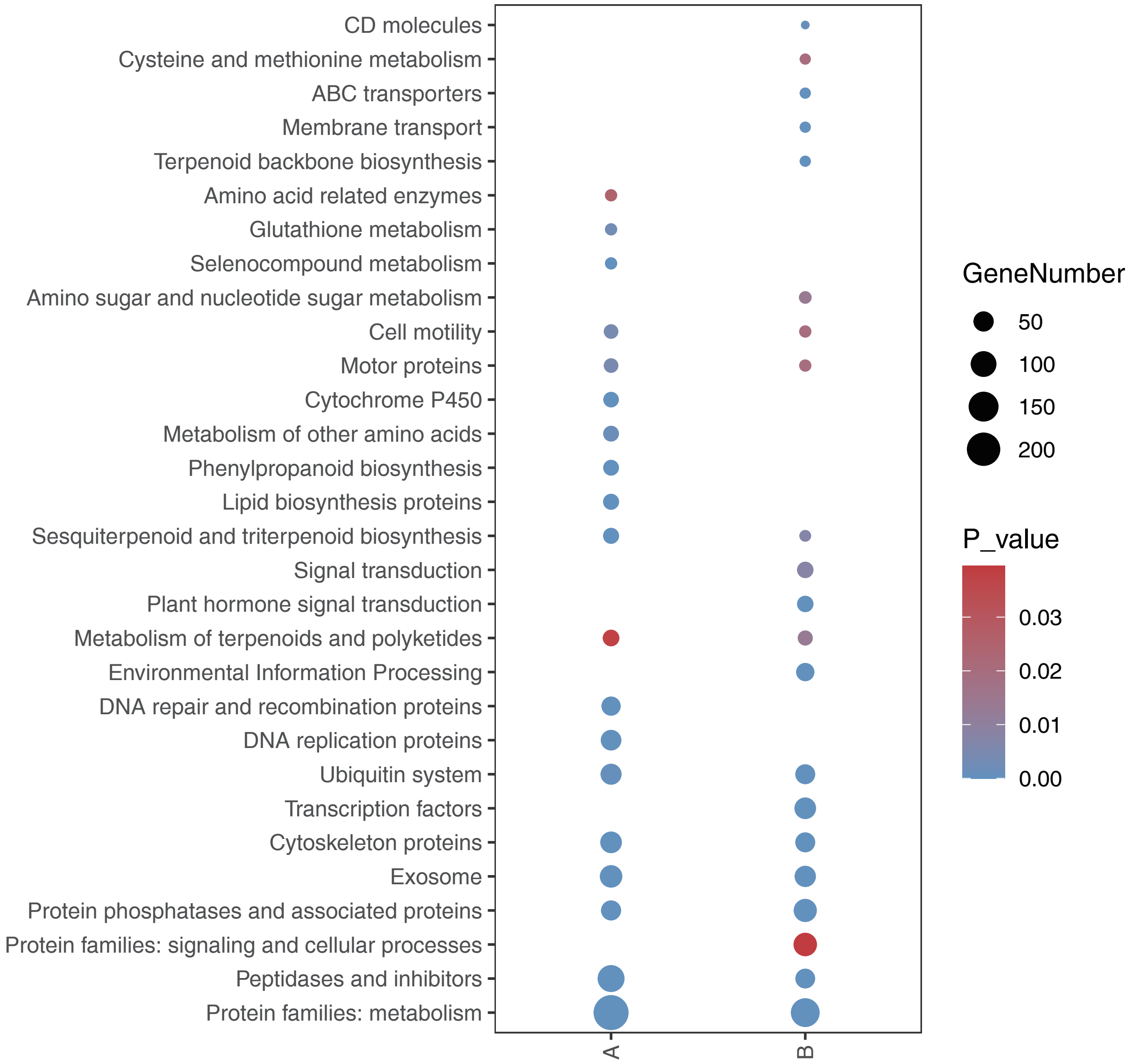

Supplementary Figure 6. Kyoto Encyclopedia of Genes and Genomes (KEGG) enrichment analyses of the expanded gene families of subgenomes A and B. The enriched terms with P-value < 0.05 are presented. Color of the bubbles indicates statistical significance of the enriched terms; size of the bubbles indicates number of genes.

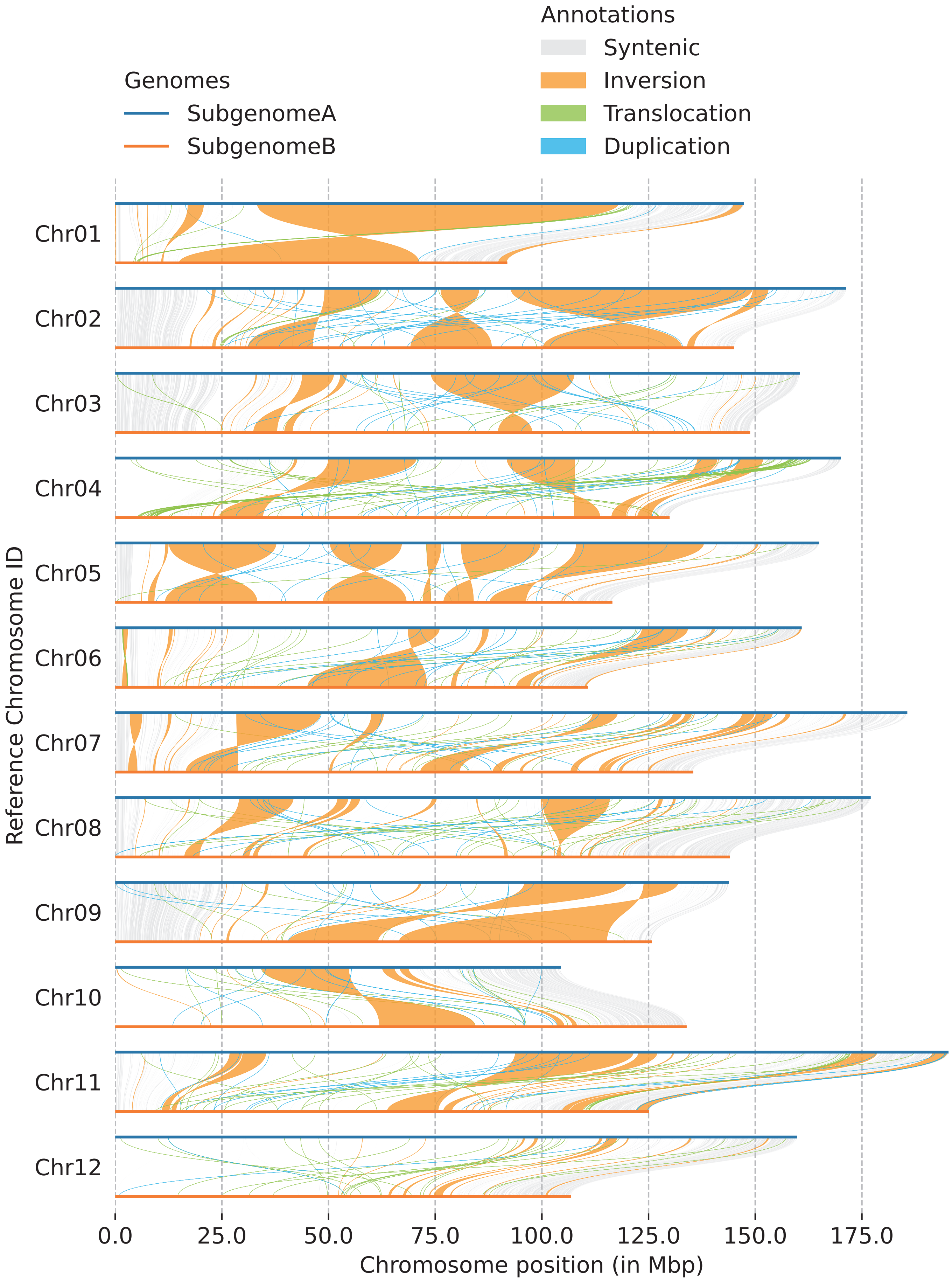

Supplementary Figure 7. Sequence collinearity among subgenomes A and B. Gray lines represent the collinearity blocks; orange lines represent the potential inversions; green lines represent the potential translocations; blue lines represent the potential duplications.

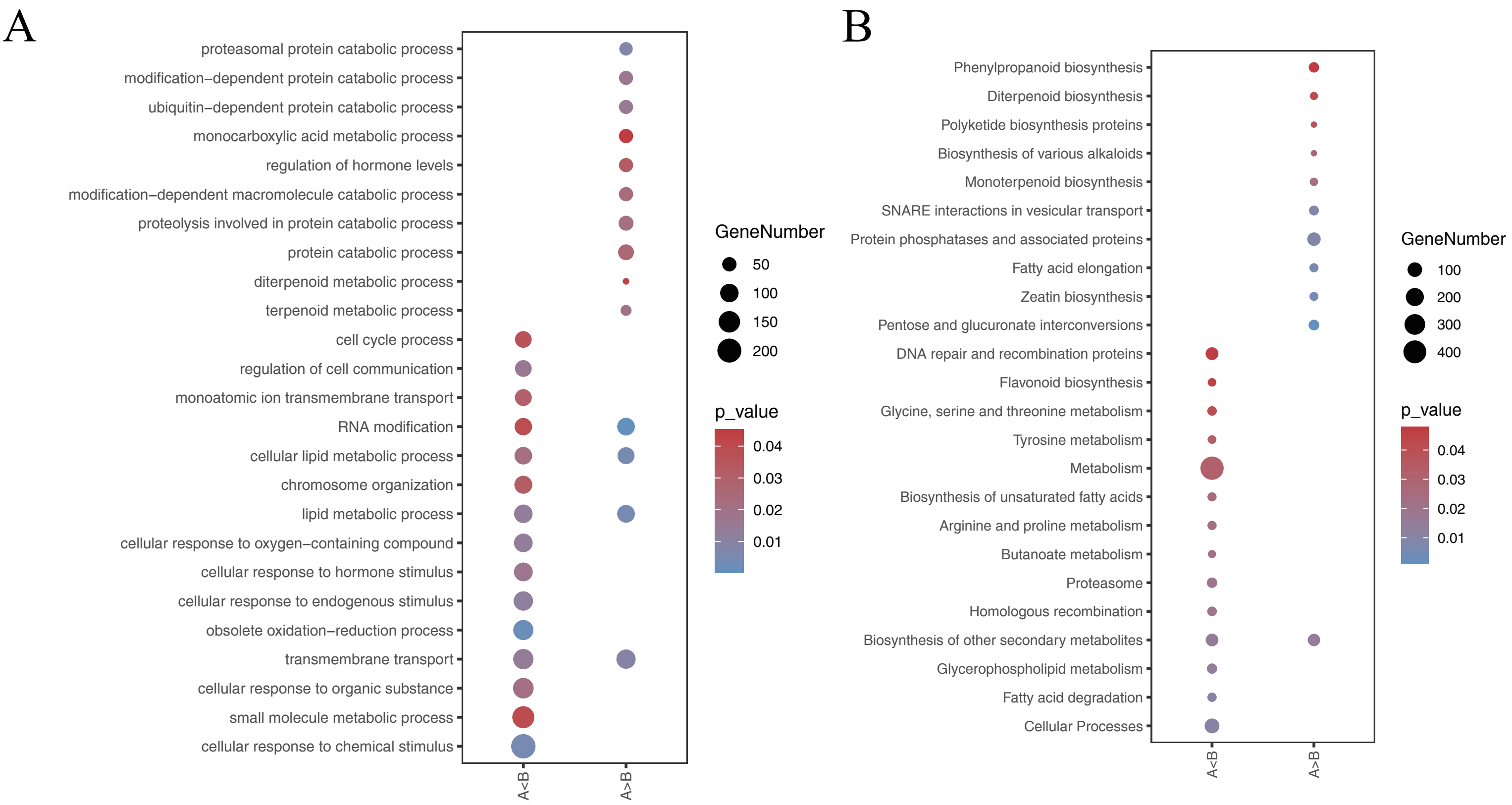

Supplementary Figure 8. (A) Significantly differentially expressed genes enrichment in Gene Ontology (GO) and (B) Kyoto Encyclopedia of Genes and Genomes (KEGG) among subgenomes A and B. The enriched terms with P-value < 0.05 are presented. The color of the bubbles indicates the statistical significance of the enriched terms; the size of the bubbles indicates the number of genes.

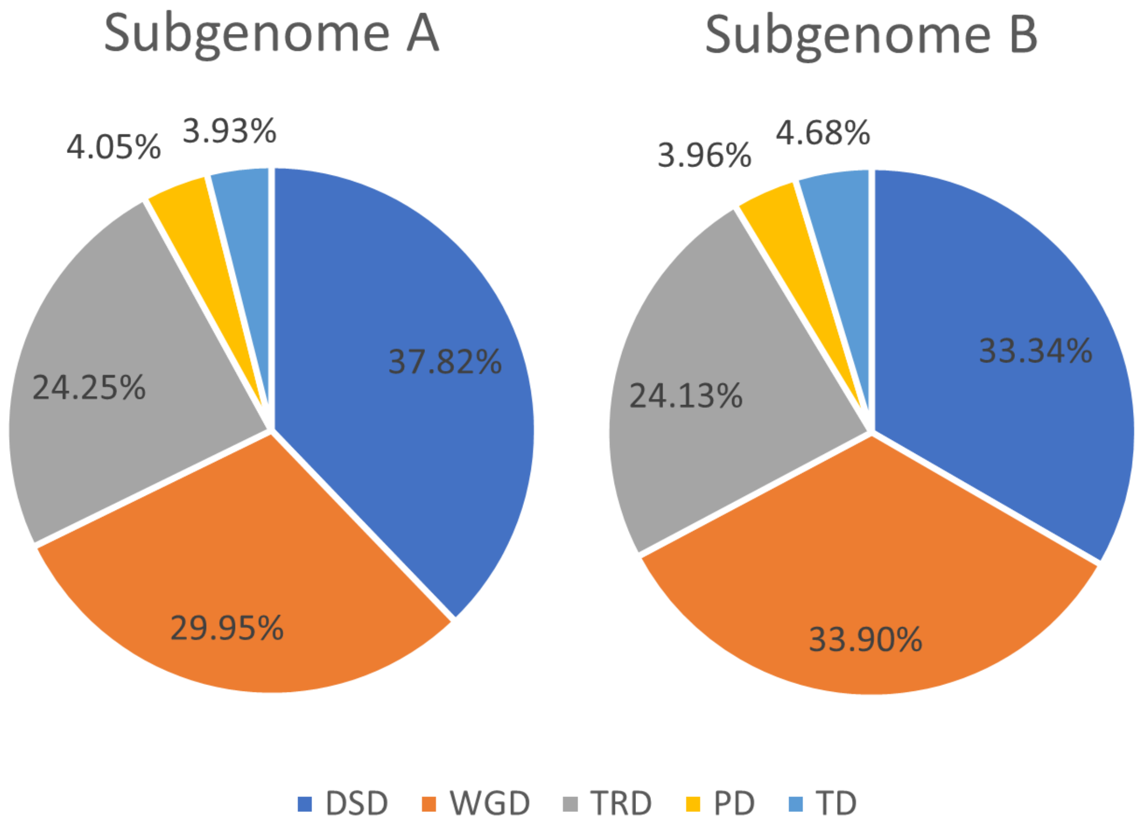

Supplementary Figure 9. Categories and proportions of different types of duplicated genes in *Panax ginseng* subgenomes A and B. DSD, dispersed duplication; WGD, whole-genome duplication; TRD, transposed duplication; TD, tandem duplication; PD, proximal duplication.

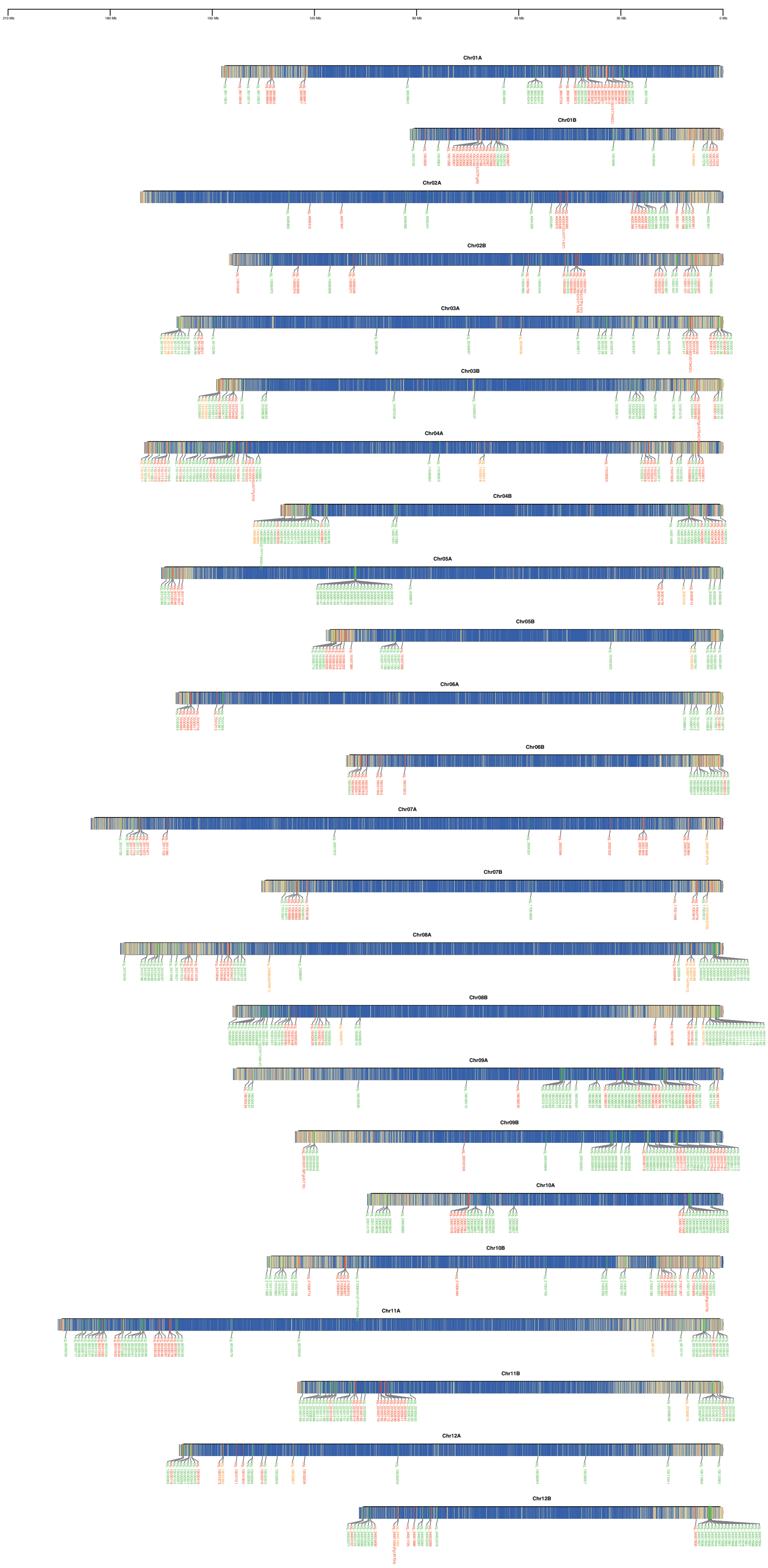

Supplementary Figure 10. Chromosomal localization of all OSC, CYP450 and UGT genes in the ginseng. The font colors represent the location of different genes, red for UGT, green for CYP450, yellow for OSC; The heat map shows the gene density, with red representing high expression and blue representing low expression.

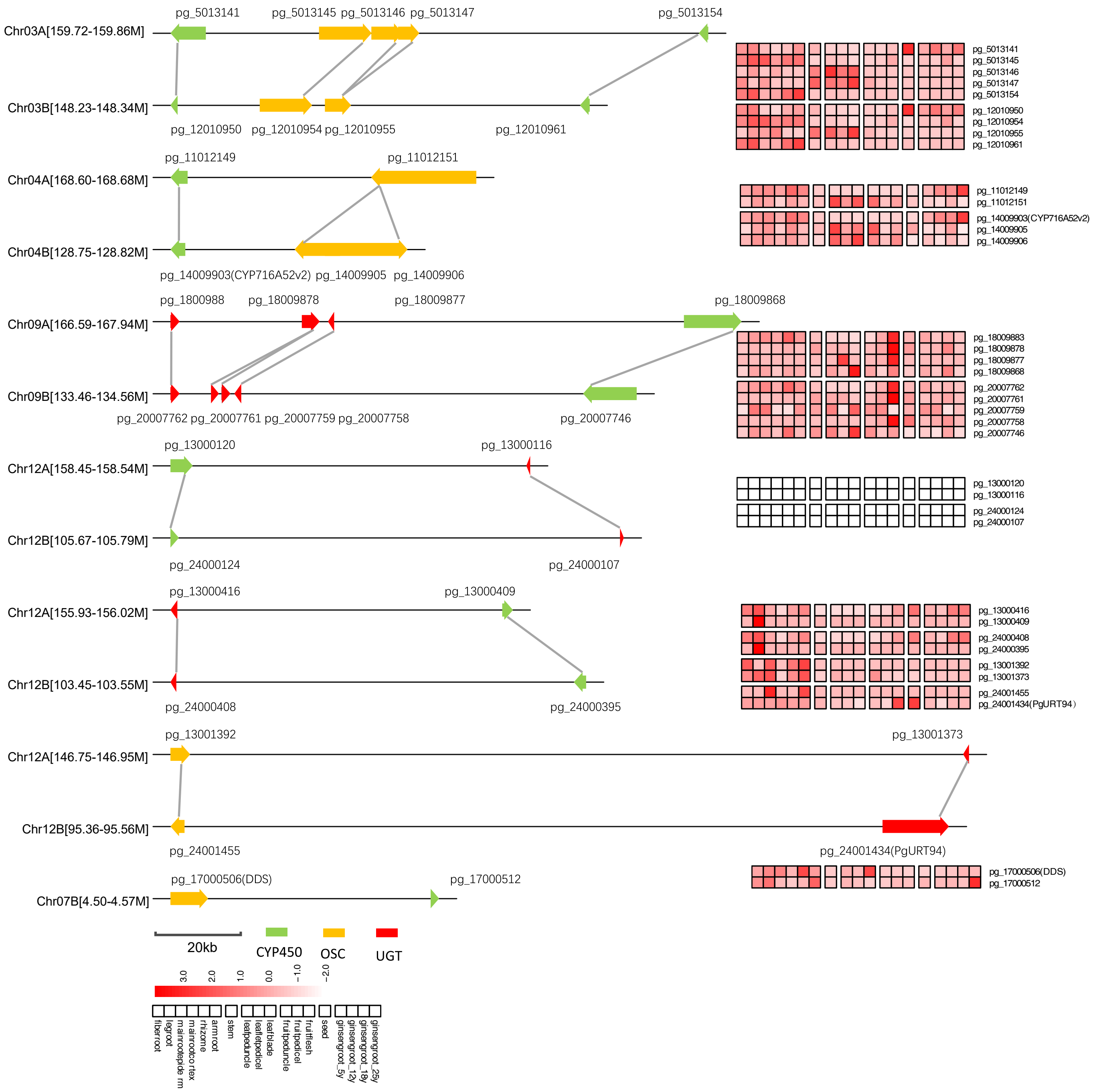

Supplementary Figure 11. Ginseng triterpenoid saponin synthesis gene cluster. UGT is glycosyltransferase; CYP450 is cytochrome P450; OSC is oxidosqualene cyclases.
